# Does sinus rhythm conversion after cardiac surgery affect postoperative health- related quality of life?

**DOI:** 10.1186/s13019-016-0459-2

**Published:** 2016-05-03

**Authors:** Henrica N. A. M. van Breugel, Orlando Parise, Fred H. M. Nieman, Ryan E. Accord, Fabiana Lucà, Pieter Lozekoot, Narendra Kumar, Ghislaine A. P. G. van Mastrigt, Jan F. M. A. Nijs, Ries Vrakking, Jos G. Maessen, Mark La Meir, Sandro Gelsomino

**Affiliations:** Department of Cardiology and Cardiothoracic Surgery, University Hospital of Maastricht, Maastricht, The Netherlands; Department of Clinical Epidemiology & Medical Technology Assessment, University Hospital of Maastricht, Maastricht, The Netherlands; Department of Cardiothoracic Surgery, Amphia Hospital Breda, Maastricht, The Netherlands; Experimental Surgery Unit, Careggi Hospital, Viale Morgagni 85, 50134 Florence, Italy

**Keywords:** Atrial fibrillation, Health related quality of life, Ablation

## Abstract

**Background:**

We investigated the impact and the predictive value of sinus rhythm at 12 months (SR_12_) on subscales of three different HrQoL questionnaires: SF-36., EuroQoL and MFI 20.

**Methods:**

Data of 125 cardiac surgery patients with pre-operative AF from our previous randomized trial were used. Based on their rhythm outcome patients were divided in two groups: SR_12_ or AF at 12 months follow up (non-SR_12_). All questionnaires were self-administered pre-operatively and at 3 months, 6 months and 12 months after surgery.

**Results:**

Synus rhytm at 12 months was predictive of improvement of SF36- mental score (MS, *p* = 0.021), Euro-QoL-MS (*p* = 0.009), VAS (*p* = 0.006), and MFI 20-MS (*p* = 0.009). We failed to find any significant interactions between SR_12_ and any of the other significant risk factors: age <65 years, paroxysmal type of AF and preoperative AF duration <12 months. In contrast, SR_12_ was not significant in predicting physical score (PS) subscales of any of the questionnaires (all, *p* > 0.05) which were predicted by age <65 years (SF36-PS, *p* = 0.029) by paroxysmal type of AF and age <65 years (Euro-QoL-PS, *p* = 0.017 and *p* = 0.04, respectively) and by AF duration <12 months, paroxysmal type of AF and age < 65 years (MFI 20-PS, *p* = 0.019, *p* = 0.020 and *p* = 0.015, respectively).

**Conclusions:**

Specific mental-related HrQoL scales are much more sensitive to sinus rhythm conversion. Sinus rithm mantainance shows significant effects on mental scores independently of other cofactors. Successful conversion to sinus rhythm after surgical ablation during cardiac surgery does not significantly affect phisical health related quality of life during 1 year follow up.

## Background

Atrial fibrillation (AF) is the most common cardiac rhythm disorder seen in the clinical practice accounting for approximately one-third of hospitalizations or cardiac rhythm disturbance [1].

Health-related quality of life (HrQoL) of patients with AF has been reported to significantly worsen, therefore enhancing HrQoL has gradually been established as one of the most important target when treating patients with AF [2]. Indeed, HrQoL has been shown to improve following medical theraphy and transcatheter ablation [3, 4]. In addition, it has beed demonstrated that conversion to sinus rhythm by surgical ablation during other cardiac surgery procedures (add-on surgery) can significantly improve the health-related quality of life [5, 6].

Nonetheless, concerns remain regarding whether this improvement in HrQoL is affected more by the treatment of the underlying heart disease than by the restoration of sinus rhythm (SR) since the relationship between conversion to SR and HrQoL has not been rigorously tested.

Therefore the present study was aimed at investigating the relationship between successful SR conversion and 1-year postoperative HrQoL after add-on surgery measuring the impact and the predictive value of SR_12_ on subscales of three different HrQoL questionnaires: SF-36., EuroQoL and MFI 20.

## Methods

### Study population

Pre- and postoperative clinical data were used from the patients enrolled in the ASAF trial [7]. Based on their rhythm outcome patients were divided in two groups: SR at 12 months follow up (SR_12_) or AF at 12 months follow up (non-SR_12_). Antiarrhythmic and anticoagulant protocols were as previously reported [7]. Patients were seen at the outpatient clinic 3 months, 6 months, and 12 months after the surgical procedure, and annually thereafter. All subjects reached 12-month follow-up. At each follow-up visit, patients underwent an electrocardiogram and, at the 12-month appointment, all patients had a 24-h Holter.

### Quality of life questionnaires

For HrQoL assessment both generic- and disease-specific questionnaires were used.

The RAND 36-item Health Survey 1.0 (SF-36) displays 8 multi-item scales (Body Pain, BP; General Health, GH, Mental Health, MH; Phisical Functioning PF; Role Emotional, RE, Role Phisical,RP; Social Functioning, SF; Vitality Vt). Each domain comprises a 5-point Likert scale ranging from 1 (bad perceived health) to 5 (excellent perceived health) [8]. SF-36 also assesses 2 major health concepts, physical and mental, with 2 composite scores, the physical composite score (PCS) and the mental composite score (MCS). The physical scales (Physical Functioning, Role Physical, Bodily Pain, and General Health) make up the PCS, and the remaining 4 scales (Mental Health, Role Emotional, Vitality, and Social Functioning) make up the MCS. Reliability by the test–retest6 between baseline and at follow-up was good, with interclass correlation coefficients ranging between 0.75 and 0.88. Internal consistency [9] was good for the 8 scales, with Cronbach coefficients ranging between 0.86 and 0.93.

The EuroQoL is a self-report questionnaire that consists of 2 parts- EQ-VAS (visual analogue scale of 0 to 100 for recording an individuals’ rating of their current health-related QoL state), and the three-level, five-dimensional descriptive system (EQ-5D), which evaluates mobility (M), self-care (SC), usual activities (UA), pain/discomfort (P/D) and anxiety/depression (A/D) on a 0 to 100 scale using the European value set [10].

The Multidimensional Fatigue Inventory (MFI-20) consists of 5 scales of fatigue (General Fatigue, GF; Mental Fatigue, MF; Phisical Fatigue, MF; Reduced Activities, RA, Reduced Motivation, RM) with scores ranging from ‘definitely tired’ to ‘definitely not tired’ on a 5-point Likert scale [11].

All questionnaires were self-administered pre-operatively and at 3 months, 6 months and 12 months after surgery.

### Statistical analysis

The power analysis was determined by Graph Pad Stat Mate software, release 2.00 (Graph Pad Prism Software, Inc, San Diego, CA) on the basis of the following assumption: type I error of 0.05 (two-sided) and difference in 12-month SF-36-mental health of 3.4. The calculated statistical power was 0.85 with a sample size per group of 62. Continuous data were expressed as mean ± standard deviation, frequence as percentage and non-normally distributed data as median and interquartile range (IQR). Data were compared for statistical significance using *t*-test, *χ*^2^ or Mann-Whitney tests as appropriate. Multiple comparisons of HrQoL scores over time were carried by ANOVA with Friedmann test.

A generalized linear model (GLM) was employed to study the the effect of conversion to SR at 12 months as well as other categorical predictors (factors) or continuous predictors (covariates) on HrQoL. More specifically, six composite HrQoL outcomes were defined : the two SF-36 composite scores (PCS and MCS), the VAS score, two EuroQoL composite scores (one mental score [EuroQoL-MS] including anxiety/depression and self-care and one physical [EuroQoL-MS] with mobility, usual activities and pain/discomfort) and two MFI20 composite scores (one mental score [MFI20-MS] including Mental Fatigue and Reduced Motivation and one physical [MFI 20-PS] with General Fatigue, Phisical Fatigue, and Reduced Activities).

All these outcomes showed a skewed distribution using histograms paneled by single outcome. The following variables were entered as potential predictors into the model: preoperative (age, gender, type of AF, AF duration, left atrial dimension, left ventricular ejection fraction, myocardial infarction,hypertension, diabetes, renal disease, chronic obstructive pulmonary disease, renal insufficiency) intraoperative (coronary artery bypass grafting, valve replacement, associated intervention,other cardiac surgery) and postoperative (SR_D_, SR_3_, SR_6_, SR_12_). On the basis of our previous experience [7] we created four new categorical variables (0/1) in the dataset: female sex, age ≥ 65 years, AF duration ≥12 months and type of AF > paroxysmal. We choosed a γ distribution with a log-link function on the basis of the best goodness-of-fit and the type III analysis. The Wald *χ*^2^, the exp(β)-Odd ration and the 95 % Wald confidence for exp(β) were reported.

SPSS v.18.0 (IBM Corp., Armonk, NY, USA) was used for analysis and a *p* value <0.05 was considered to indicate statistical significance.

## Results

### Patient characteristics and rhythm follow up

Seven patients died during the 12 months follow up therefore a total of 125 patients were taken into account for analysis. Among them, sixty-two (47 %) were in SR sinus rhythm at 12 months follow up whereas 63 (53 %) had recurrent arrhythmia. Table 1 dysplays patients characteristics. Subjects who were not in SR at 12 months had larger atria (*p* < 0.001) and an higher perentage of patients with permanent AF (<0.001). No other difference was detected between groups.

**Table 1:** Demographic Data (*n* = 125)

	Study population	SR12-group (*N* = 62)	Non-SR12-group (*N* = 63)	*p*-value
Age (Years)	67.9 ± 8.9	67.4 (39.0–84.0)	68.4 (46.0–81.0)	.38
Weight (Kg)	79.2 ± 17.0	78.6 (49–170)	79.7 (53–111)	.73
Gender (male)	79 (63.2 %)	42 (66.7 %)	37 (59.7 %)	.42
*Civil state*				.16
Divorced	8 (6.5 %)	7 (11.1 %)	1 (1.6 %)	
Married	84 (87.7 %)	42 (66.7 %)	42 (68.9 %)	
Unmarried with partner	10 (8.1 %)	5 (7.9 %)	5 (8.2 %)	0.16
Widow(er)	22 (17.7 %)	9 (14.3 %)	13 (21.3 %)	
*Education*				
Primary school	27 (22.0 %)	11 (17.5 %)	16 (26.7 %)	
Lower education	30 (24.4 %)	15 (23.8 %)	15 (25.0 %)	.52
Intermediate education	45 (36.6 %)	24 (38.1 %)	21 (35.0 %)	
High education	21 (17.1 %)	13 (20.6 %)	8 (13.3 %)	
*Previous cardiac history*				
Atrial Fibrillation:				
Paroxysmal AF	54 (43.2 %)	37 (58.7 %)	17 (27.4 %)	
Permanent AF	41 (32.8 %)	11 (17.5 %)	30 (48.4 %)	< .01
Persistent AF	28 (22.4 %)	13 (20.6 %)	15 (24.2 %)	
Atrial flutter	2 (1.6 %)	2 (3.2 %)	0 (0 %)	
Total months of AF	82.0 ± 104.8	67.9 (3–403)	96.9 (3–617)	.03
Left Atrial Dimension (mm)	50.6 ± 7.4	48.7 (33–65)	53.3 (40–70)	<.01
Left Ventrical Ejection Fraction (%)	52.6 ± 13.5	53.2 (18–80)	51.9 (22–75)	.68
Co-morbidity/Riskfactors (*N* = 122)				
Hypertension	51 (41.8 %)	25 (40.3 %)	26 (43.3 %)	.74
Stroke	6 (4.9 %)	2 (3.2 %)	4 (6.7 %)	.38
Smoking:				.50
Never	56 (46.3 %)	30 (48.4 %)	26 (44.1 %)	
Current	16 (13.2 %)	6 (9.4 %)	10 (16.9 %)	
Past	49 (40.5 %)	26 (41.9 %)	23 (39.0 %)	
Positive Family History	61 (49.2 %)	27 (42.9 %)	34 (55.7 %)	.15
Diabetes	18 (14.8 %)	13 (21.0 %)	5 (8.3 %)	.05
Renal Disease	7 (5.6 %)	3 (4.8 %)	4 (6.5 %)	.68
COPD	20 (16.3 %)	8 (12.7 %)	12 (20.0 %)	.27
Previous myocardial infarction	31 (24.8 %)	19 (30.2 %)	12 (19.4 %)	.16
Cardiomyopathy	21 (17.1 %)	10 (16.1 %)	11 (18.0 %)	.78
Congenital Heart disease	7 (5.7 %)	2 (3.2 %)	5 (8.3 %)	.22
Systolic Blood Pressure (mmHg)	141.9 ± 26.6	144.7 (70–200)	139.3 (80–190)	.28
Diastolic Blood Pressure (mmHg)	79.4 ± 17.8	82.3 (40–180)	76.5 (40–110)	.18
*Pre-operative complaints (N = 123)*				
Palpitations	51 (41.5 %)	28 (45.2 %)	23 (37.7 %)	.40
Dyspnea	98 (79.7 %)	48 (77.4 %)	50 (82.0 %)	.53
Angina	49 (40.2 %)	24 (39.3 %)	25 (41.0 %)	.85
(Pre-) Syncope	7 (5.7 %)	3 (4.8 %)	4 (6.6 %)	.68
Dizziness	32 (26.0 %)	17 (27.4 %)	15 (24.6 %)	.72
Fatigue	62 (50.4 %)	31 (50.0 %)	31 (50.8 %)	.93
Other complaints	12 (9.8 %)	5 (8.1 %)	7 (11.7 %)	.50
*Operative data (N = 125)*				
Coronary Artery Bypass Grafting	39 (31.2 %)	23 (36.5 %)	16 (25.8 %)	
Valve replacement	50 (40.0 %)	24 (38.1 %)	26 (41.9 %)	.11
CABG and Valve replacement	28 (22.4 %)	15 (23.8 %)	13 (21.0 %)	
Other cardio-surgery	8 (6.4 %)	1 (1.6 %)	7 (11.3 %)	

*Abbreviations*: *SR* Sinus Rhythm, *AF* Atrial Fibrillation, *COPD* Chronic obstructive pulmonary disease, *CABG* Coronary artery bypass graft

### Morbidity and mortality

No significant differences were found between SR-group and non-SR-group regarding the number of rethoracotomies (*p* = 0.56), pulmonary complications (*p* = 0.07), myocardial infarction (*p* = 0.23), renal failure (*p* = 0.84), stroke (*p* = 0.38), wound infections and other infections (*p* > 0.9), hours of ICU stay (*p* = 0.09) and total hospital stay in days (*p* = 0.11).

### Effect of Add-on surgery on HrQoL

#### SF-36

**Fig. 1:**
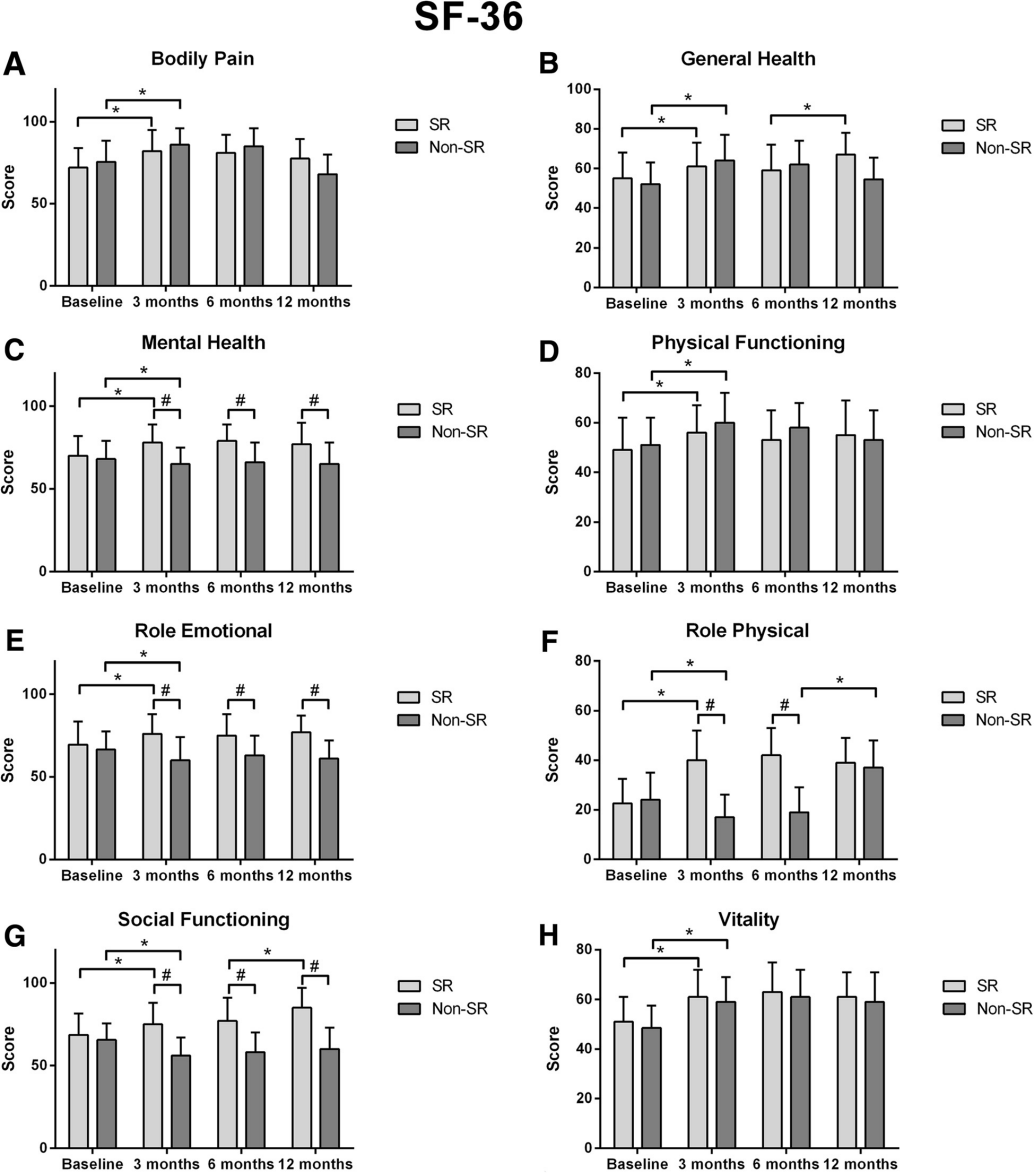
SF-36 subscales in patients with or without recurrent arrhythmia at 12 month-follow-up. ^*^ Significance between follow-up steps.^≠^ Inter-group significance significance

Figure 1 shows the comparison in SF-36 domains between patients with or without AF at different times. At baseline SF-36 scores were comparable between Groups. All subscales of SF-36 improved at 3 months in patients without arrhythmia. In contrast, in patient with recurrent arrhythmia BP, GH, PF an Vt which improved fronm baseline (*p* < 0.05), MH, RE, RF and SF worsended from baseline (all, *p* < 0.01). The latter domains were significantly lower in patient with recurrent AF (*p* < 0.05). At 6 months no significant variation was detected in both groups in SF- scales with intergroup-differences in MH, RE, RF and SF remaining unchanged (all, *p* < 0.01).

Finally, at 12 months, only SF and GH further Improved in patients in SR. In patients with AF Mental Health, RE and SF worse than in patients with no-AF. In contrast RP improved and was not significantly different compared to AF patients.

**Fig. 2:**
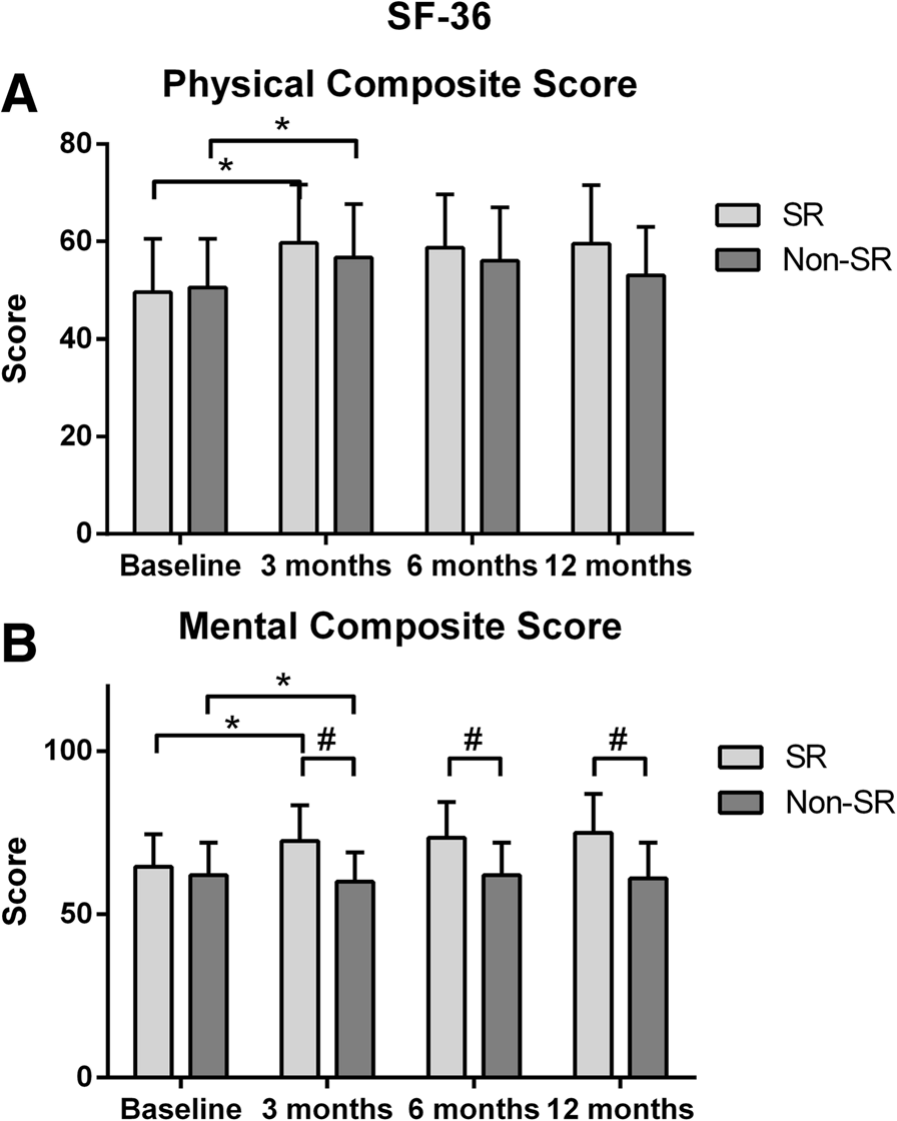
SF-36 subscales Composite Scores. in patients with or without recurrent arrhythmia at 12 month-follow-up. * Significance between follow-up steps.^≠^ Inter-group significance significance

Finally Physical composite score was not significantlty different between groups whereas mental composite score was significantly higher in patients without AF recurrence (Fig. 2).

#### EuroQoL

Compared with baseline, EQ-5D and EQ-VAS improved significantly at 12 months either in patients with SR (61.2 ± 11.3 vs. 74.0 ± 12.8, *p* < 0.001 and 67.5 ± 10.9 vs. 71.0 ± 12.0, *p* = 0.004) or those with AF (61.3 ± 10.7 vs. 71.0 ± 12.3, *p* = 0.003 and 68.1 ± 10.3 vs. 72.0 ± 11.4, *p* = 0.02) with no significance between Groups (*p* = 0.06 and *p* = 0.79 for EQ-5D and EQ-VAS, respectively.

**Fig. 3:**
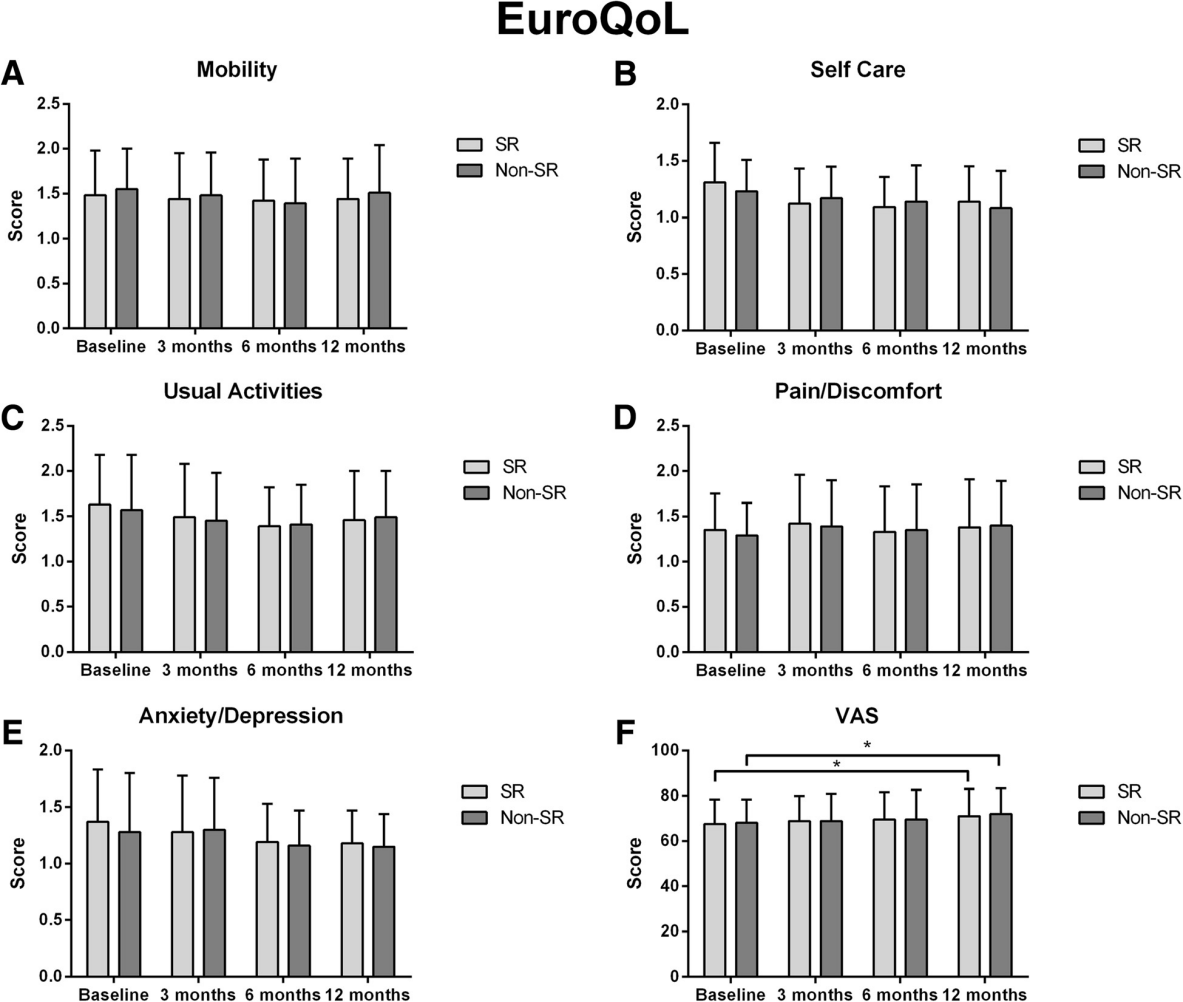
EQ-5D sub-domains and VAS score in patients with or without recurrent arrhythmia at 12 month-follow-up. * Significance between follow-up steps.^≠^ Inter-group significance significance

Figure 3 shows that no difference was found in any EQ-5D sub-domains at 3, 6 or 12 months.

#### MFI-20

**Fig. 4:**
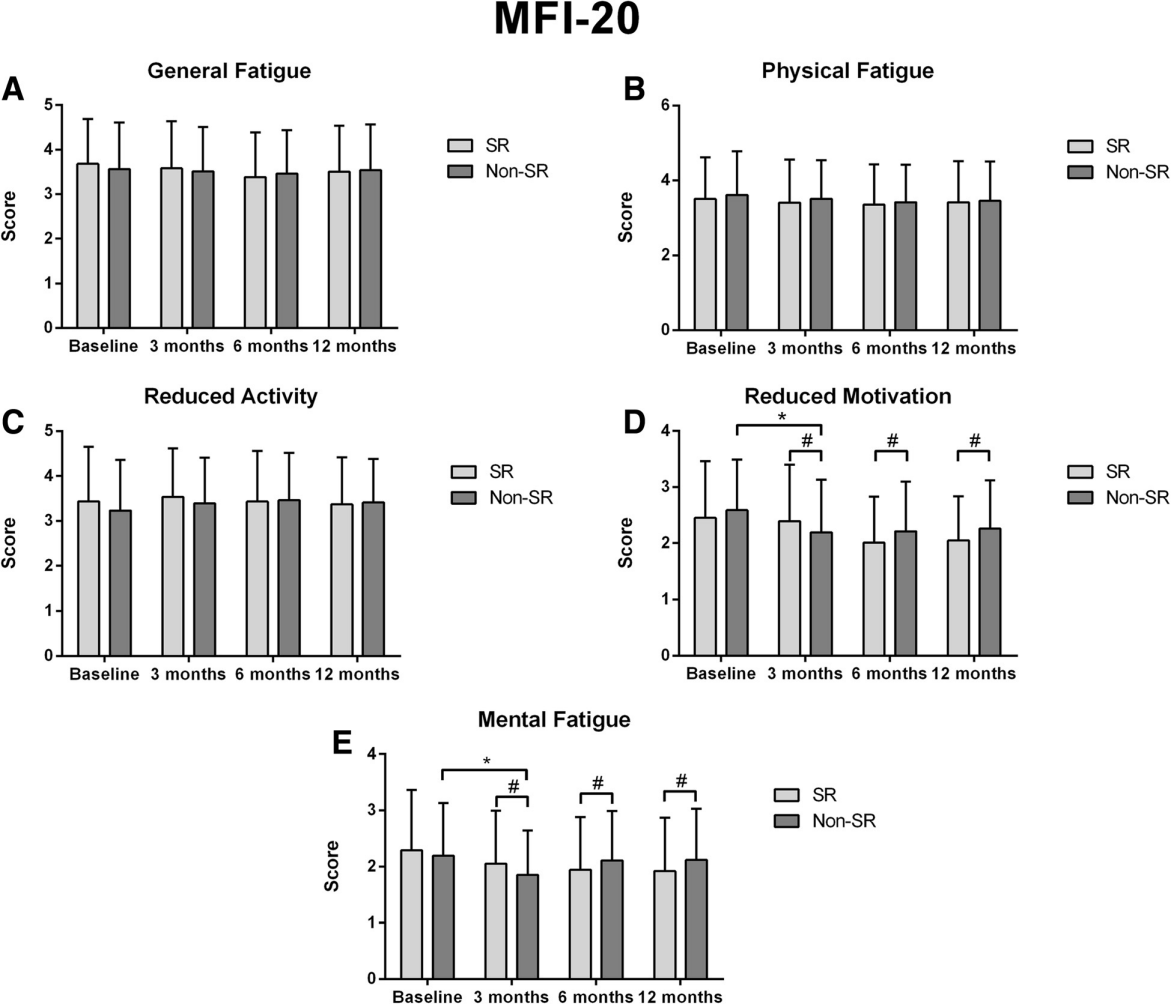
MFI-20 sub-domains in patients with or without recurrent arrhythmia at 12 month-follow-up. * Significance between follow-up steps.^≠^ Inter-group significance significance

At 3 months, changes in GF, PF and RA were not significant in both group with any difference detected between patients with or without AF recurrence (Fig. 4). In contrast, in patients no in sinus rhythm MF and RM were significantly lower compared to baseline (*p* = 0.004) and to no-AF patients (*p* < 0.001). At 6 months and 12 months MF and RM did not improve in patient with AF and both these domains were significantly higher than SR patients (*p* < 0.001 at 6 and 12 months). No other relevant changes were detected in patients in SR at 6 and 12 months.

### Relationship between 12-month SR and HrQoL

Results of GLM are shown in Table 2. Synus rhytm at 12 months was independent predictor of SF36-MS. Age <65 years and preoperative AF duration <12 months were also significant. SR_12_ also predicted Euro-QoL-MS (with paroxysmal type of AF), VAS and MFI 20-MS.

**Table 2:** Parameters estimates

	Wald *χ* ^2^	*p*	Exp β	95 % CI Exp β
SF 36 –MS				
SR_12_	9.235	0.021	0.892	0.753–0.995
Age < 65 yrs.	7.818	0.018	1.211	1.098–1.431
AF duration < 12 months	8.190	0.019	0.965	0.801–1.104
SF 36 –PS				
Age < 65 yrs.	6.542	0.029	1.156	0.945–1.301
Euro QoL-MS				
SR_12_	8.221	0.009	0.788	0.589–0.873
AF Paroxysmal	10.234	0.005	1.167	0.902–1.298
Euro QoL-PS				
Age < 65 yrs.	7.892	0.017	1.115	0.953–1.199
AF Paroxysmal	10.003	0.004	1.056	0.862–1.189
Euro QoL-VAS				
SR_12_	9.776	0.006	0.876	0.678–0.942
AF Paroxysmal	9.993	0.008	1.055	0.899–1.178
MFI 20-MS				
SR_12_	8.569	0.009	1.188	0.976–1.244
Age < 65 yrs.	7.908	0.016	1.045	0.932–1.177
MFI 20-PS				
Age < 65 yrs.	9.044	0.019	1.266	1.088–1.399
AF Paroxysmal	9.302	0.020	0.877	0.745–0.978
AF duration < 12 months	8.805	0.015	0.915	0.802–1.105

*Abbeviations*: *CI* Confidence Interval, *MS* Mental Score, *PS* Physical Score, *AF* Atrial Fibrillation

In contrast, SR_12_ was not significant for SF36-PS, Euro-QoL-PS and MFI20 –MS (all, *p* > 0.05).

On the contrary SF36-PS was predicted by age <65 years, Euro-QoL-PS by age <65 years and paroxysmal type of AF whereas MFI 20-PS by age <65 years, paroxysmal type of AF and preoperative AF duration <12 months. SR_3_ and SR_6_ resulted to be not significant.

**Fig. 5:**
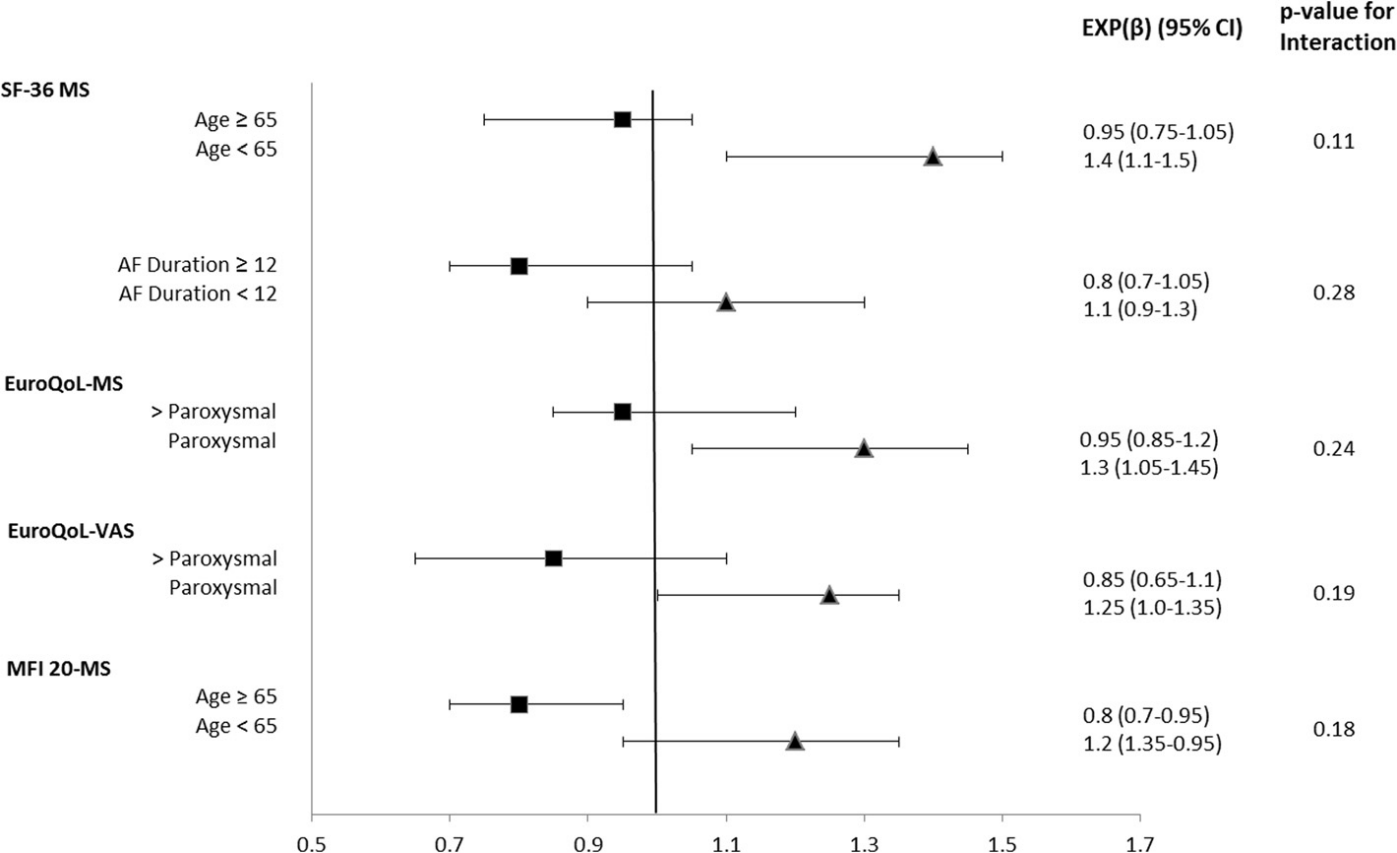
Sub-analysis analysis of effect of SR_12_ on HrQoL- questionnaires by co-factors

The subanalysis of the influence of SR_12_ according to the presence –absence of other cofactors showed that patients in SR at 12 months were associated with increased HrQoL regardless of age, preoperative AF durationand type of AF. There were no apparent significant interactions between SR_12_ and any of the covariates (Fig. 5).

## Discussion

At the best of our knowledge, this is the first study to explore the relationship between manteinance of sinus rhythm 12 months after surgical ablation (SR_12_) associated to other cardiac procedures (add-on surgery) and health-related quality of life (HrQoL). The purpose of the present report was to measure the impact and the predictive value of SR_12_ on subscales of three different HrQoL questionnaires: SF-36, EuroQoL and MFI 20.

The main findings of our study can be summarized as follows: First, sinus rhythm at 12 months was predictive of improvement of SF36-MS, Euro-QoL-MS, VAS and MFI 20-MS. Furthermore, when we allowed for interaction between type SR_12_ and other significant risk factors (age <65 years, paroxysmal type of AF and preoperative AF duration <12 months) we found that the effect of SR_12_ on SF36-MS, Euro-QoL-MS, VAS and MFI 20-MS was similar or even higher in low- versus high-risk- patients. Moreover, we failed to find any significant interactions between SR_12_ and any of the other risk factors demonstrating that SR_12_ remains a critical determinant of these HrQoL outcomes beyond any other potentially associated predictor.

It has been demonstrated that the impact of AF on HrQoL extends beyond symptoms. Indeed, the psychological and emotional effects of AF can be debilitating and several limited studies suggest that psychological distress may be linked with patient-reported AF symptom severity [12] and that patients with AF have a high prevalence of anxiety and depression [13, 14]. Furthermore, symptoms of depression and anxiety have been shown to be strong predictor of worsened quality of life [15, 16] and worsened outcomes [17–20] in patients with AF. However, there are limited data on the association fo SR manteinance and psychological status. Our study demonstrated the important influence of SR on HrQoL-mental scores regardless of which specific scale was used. In other words,in case of recurrent AF after surgery, psychological comorbidities may heavily influence healthcare consumption. This aspect warrants further larger studies.

Second, SR_12_ was not significant in predicting SF36-PS, Euro-QoL-PS and MFI20 -PS (all, *p* > 0.05).

Our findings are in accordance with data from a recent reasearch [21] showing that patients who maintained sinus rhythm after ablation had a significant improvement in AF symptoms and HrQoL whereas no improvement was observed in patients with recurrent AF.

Prior studies for largely paroxysmal AF treated with catheter ablation showed a significant post-ablation amelioration of HrQoL, with the benefit lasting over 2 years [22, 23]. Furthermore, Fiala et al [24] found a significant rise in QoL after catheter ablation of long-standing persistent AF at 1 year that further slightly increased at 2 years. Nonetheless, these authors, unlike for functional benefits, were not able to demonstrate a significant difference between SR and non-SR subgroups at 1 year when modest HrQoL improvement was also present in patients examined in AF/AT.

Third, in our experience SF36-PS was predicted by age <65 years, Euro-QoL-PS by age <65 years and paroxysmal type of AF whereas MFI 20-PS by age <65 years, paroxysmal type of AF and preoperative AF duration <12 months.

It is not surprising that patients with AF > paroxysmal show worse physical HrQoL subscales but it is interesting that SR_12_ was not predictive of pysical improvement despite patients in AFat 12 months a significantly higher percentage of pemanent AF patients (48.4 % vs. 17.5 %). This suggest that the AF type is a primary predictor and it is not strenghten by the postoperative rhytm. This was confirmed by the not significant interaction between the two variables (*p* = 0.44).

Regarding age,as we might expect, ≥ 65 years showed a worse HrQoL after surgery. However, it must be considered that elderly patients differ considerably from patients in the younger age group as they have a higher incidence of AF associated with other risk factors and frequent multiple comorbidities which may affect their pst-surgery HrQoL. Nonetheless, we do believe that there is no reason to deny a priory add- on surgery to elderly since results from the literature suggest that catheter ablation of AF in elderly patients can be performed with success rates comparable to those in younger without an increase in complication rate [25].

### Study limitations

Our study has some limitations which need to be highlithed.

First of all, this study was performed retrospectively and this could have significantly affected our results. Second,we employed an old version and no disease specific-HrQoL questionnaires. It is well known that HrQoL outcomes can be weakened by other life events or physical complaints during follow up. This ‘attenuation’ effect may become even larger as the follow up period extends.

## Conclusions

Specific mental-related HrQoL scales are much more sensitive to sinus rhythm conversion. Sinus rithm mantainance shows significant effects on mental scores independently of cofactors such as age, type of AF and AF type. Successful conversion to sinus rhythm after surgical ablation during cardiac surgery does not significantly affect phisical health related quality of life during 1 year follow up.
